# Rhabdomyolysis secondary to Influenza A infection in a patient using antipsychotic and serotonergic agents: A case report

**DOI:** 10.1177/2050313X251392105

**Published:** 2025-11-01

**Authors:** Rahul S. Nanduri, Noah Karnath, Alekhya Gurram, Arkoon Ali, Bernard Karnath

**Affiliations:** 1 John Sealy School of Medicine, University of Texas Medical Branch, Galveston, USA; 2 Department of Internal Medicine, University of Texas Medical Branch, Galveston, USA

**Keywords:** rhabdomyolysis, acute kidney injury, Influenza A, case report, creatine kinase

## Abstract

Influenza A infection is most commonly associated with pulmonary disease. However, viral infection can also rarely induce extrapulmonary complications, such as myocarditis, neuropsychiatric pathologies, and rhabdomyolysis. Influenza-A-induced rhabdomyolysis is an uncommon complication, and failure to appropriately address this pathology can quickly lead to deterioration into acute kidney injury. Recognizing this clinical presentation and differentiating it from viral myalgias is often difficult, as muscle aches are a common feature of Influenza A infection. This challenge is further exacerbated by the broad differential of rhabdomyolysis, including various medications such as antipsychotic and serotonergic medications. We report a case of an adult taking risperidone and trazodone presenting with profound myalgias and dark urine following Influenza A viral illness, who was found to have creatine kinase levels exceeding 60,000 U/L. We highlight relevant literature, discuss various proposed pathophysiological mechanisms, and highlight appropriate management strategies. Fluid resuscitation is a mainstay of management; however, iatrogenic fluid overload, such as pulmonary edema, is a possible complication that must be appropriately addressed. This case underscores the importance of maintaining a high index of suspicion for rhabdomyolysis in patients with Influenza A and presents a novel narrative of rhabdomyolysis resulting from a potentially additive interplay of pharmacological (use of trazodone and risperidone) and nonpharmacological predisposing factors.

## Introduction

Influenza A is a negative-sense ribonucleic acid (RNA) virus in the Orthomyxoviridae family.^1^ Of three subtypes (A, B, and C), Influenza A is the most susceptible to antigenic variation, causing it to be highly contagious.^1^ Cardinal symptoms include acute-onset fever, chills, cough, myalgias, and malaise. Although Influenza A is usually linked to pulmonary disease, infection has been increasingly associated with extrapulmonary complications, including rhabdomyolysis.^2^

Rhabdomyolysis is characterized by skeletal muscle injury, releasing toxic contents that promote acute kidney injury.^3^ Rhabdomyolysis can be caused by trauma, such as crush injury or compartment syndrome, or non-traumatic causes, such as hyperthermia, ischemic damage, metabolic deficiencies, medication-induced adverse effects, and infectious causes. With respect to medications, risperidone and trazodone, among others, are both well-known to have rhabdomyolytic potential. Of infectious causes, several bacteria (such as Legionella and Salmonella), protozoa, fungi, and viruses have been identified as triggering rhabdomyolysis.^4^ Of viral causes, Influenza A is the most common trigger as compared to COVID-19, Coxsackieviruses, and Human Immunodeficiency Virus.^4^

Reports of viral rhabdomyolysis in patients taking rhabdomyolytic medications are scarce, limiting the exploration of a possible additive interplay of infectious and pharmacological predisposing factors. To our knowledge, we present the first case of Influenza A-induced rhabdomyolysis in a patient taking risperidone and trazodone and discuss the clinical course along with management strategies.

## Case

The patient is a 71-year-old male with a history of paranoid schizophrenia who presented to the emergency department with generalized weakness and dark urine. Social history included a 40 pack-year smoking history, and medications included risperidone 3 mg twice daily and trazodone 50 mg daily with good compliance for over a decade. Family history was noncontributory. Two days prior, he noted an unproductive cough, flu-like symptoms, and shortness of breath, with no relief from cough syrup or ibuprofen. One day later, the patient noticed progressive myalgias, darkened urine, and extreme bilateral lower extremity weakness, causing a fall with trauma to both knees. The patient later sustained another fall, hitting his head, after which he was brought to our institution. Upon presentation, the patient endorsed malaise, fatigue, sore throat, shortness of breath, wheezing, hematuria, and generalized weakness. He denied hemoptysis, chest pain, orthopnea, or lower extremity swelling.

The patient was admitted to the hospital, and blood pressure was 92/85, pulse was 104 bpm, respirations were 20 breaths/min, temperature was 98.9°F, and SpO2 was 93%. Physical examination was significant for a central forehead hematoma, generalized wheezing and rhonchi, and tenderness on bilateral knee flexion. CT head was negative. Influenza A testing was positive, and urinalysis suggested myoglobinuria. COVID-19, Influenza B, respiratory syncytial virus (RSV), and Hepatitis A, B, and C testing returned negative. Labs revealed creatine kinase (CK) level of 61,180 and mild hypokalemia. The patient was admitted with a diagnosis of rhabdomyolysis and started on oseltamivir, potassium chloride, ipratropium bromide/albuterol 0.5 mg–3 mg/3mL, and IV Lactated Ringer’s at 200 mL/h due to the markedly elevated CK level and low urine output averaging to 39 mL/h.

**Figure 1.**
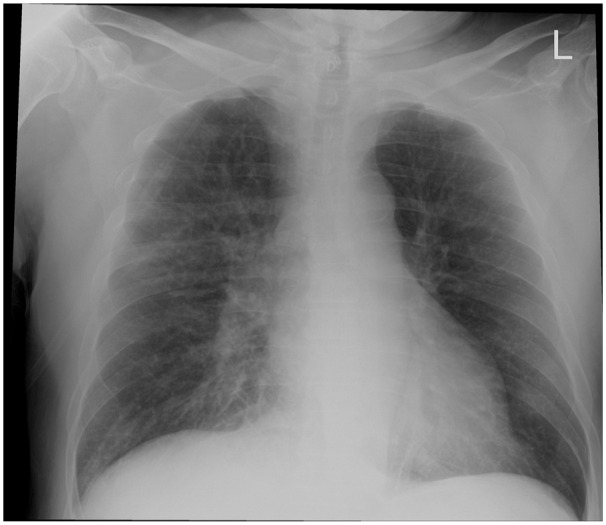
Chest X-ray showing hazy opacification of hila and central vascular congestion with prominent hazy interstitial markings suggestive of pulmonary edema.

Nephrology was consulted for fluid management on day 1 and advised titrating fluids to 125 mL/h as per the goal urine output of 200–300 mL/h. Sodium bicarbonate 650 mg twice daily was started, and calcium and potassium were repleted as needed. On day 3, urine output totaled 1.3 L over the last 24 h (average urine output of 54 mL/h). Given failure to reach the goal, urine output, IV fluids were increased to 150 mL/h. Due to diffuse wheezing, azithromycin 500 mg and prednisone 40 mg were initiated for symptomatic respiratory management. On day 4, he developed hyperkalemia of 6.0 mmol/L, and a Chest X-ray showed central pulmonary vascular congestion suggestive of pulmonary edema (Figure 1).

**Figure 2.**
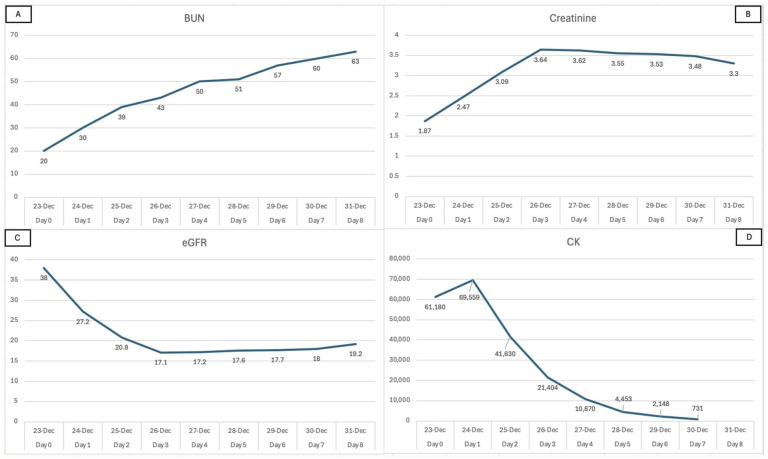
Line graphs showing trends in blood urea nitrogen (BUN) (a), Creatinine (b), estimated glomerular filtration rate (eGFR) (c), and creatine kinase (CK) (d) throughout hospitalization.

IV fluids were decreased to 100 mL/h, and furosemide, calcium gluconate, D50, insulin lispro, and sodium zirconium cyclosilicate were administered in response to the fluid-overloaded status and hyperkalemia. Potassium decreased to 4.8 on day 5, and IV fluids were halted. Repeat Chest X-ray showed a reduction in fluid congestion. Relevant lab values are shown in Table 1, and trends in Blood Urea Nitrogen, Creatinine, estimated Glomerular Filtration Rate, and CK are shown in Figure 2.

**Table 1.** Relevant lab values highlighting rhabdomyolytic renal injury and subsequent resolution.

Labs	Pre-admit	Admit	Day 4	Day 7	2 weeks post-discharge	2 months post-discharge
Hgb (12.2–16.4 g/dL)	15.7	13.6	**10.5**	**10.9**	13.0	—
Na (135–145 mmol/L)	**137**	**131**	**134**	140	136	137
K (3.5–5.0 mmol/L)	4.3	**3.4**	**6.0**	3.9	3.9	3.9
Cl (98–108 mmol/L)	104	104	**110**	107	107	107
Total CO_2_ (23–31 mmol/L)	24	**21**	**21**	31	**22**	23
Blood urea nitrogen (7–23 mg/dL)	15	20	**50**	**60**	**32**	16
Cr (0.60–1.25 mg/dL)	1.07	**1.87**	**3.62**	**3.48**	**1.85**	**1.46**
Ca (8.6–10.6 mg/dL)	8.9	**7.4**	—	**7.7**	8.8	8.6
Alk Phos (34–122 U/L)	87	89	70	—	90	—
AST (13–40 U/L)	20	**791**	**296**	—	21	—
ALT (5–50 U/L)	18	**167**	**204**	—	21	—
CK (33–194 U/L)	**—**	**61,180**	**10,870**	**731**	—	124
eGFR (90–120 mL/min/1.73 m^2^)	74.7	**38**	**17.2**	**18**	**38.5**	**51.1**
Troponin I (⩽0.034 ng/mL)	**—**	**0.120**	**—**	—	—	—
Influenza A NAAT (negative)	**—**	**Positive**	**—**	—	—	—
Urinalysis appearance (clear)	**—**	**Turbid**	**—**	—	—	—
Urinalysis color (colorless)	**—**	**Yellow**	**—**	—	—	—
Urine blood (negative)	**—**	**3+**	**—**	—	—	—
Urine protein (<20 mg/dL)	**—**	**100**	**—**	—	—	—
RBC/HPF (0–3 HPF)	**—**	1	**—**	—	—	—
WBC/HPF (0–5 HPF)	**—**	**9**	**—**	—	—	—
Bacteria (negative)	**—**	**Few**	**—**	—	—	—
Granular casts (⩽1 LPF)	**—**	1	**—**	—	—	—

WBC: white blood cell; RBC: red blood cell; HPF: high-power field; NAAT: nucleic acid amplification test; AST: aspartate aminotransferase; ALT: alanine aminotransferase; eGFR: estimated glomerular filtration rate; CK: creatine kinase.

Abnormal values in bold text.

**Figure 3.**
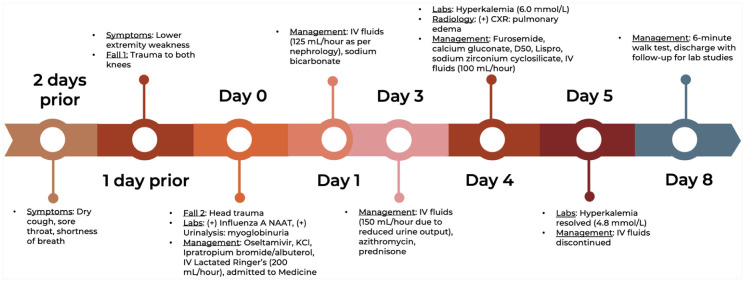
Clinical timeline illustrating the patient’s disease course and notable events throughout the hospitalization.

Creatine and eGFR levels stabilized after day 3, while the CK level steadily decreased. BUN level continued to rise, but at a much slower rate after day 3. The patient exhibited good response to furosemide challenges on days 6 and 7, although the patient failed a 6-min walk test on day 7. On day 8, the patient was discharged after passing the 6-min walk test. The clinical timeline is summarized in Figure 3.

Throughout the hospital stay, the need for renal replacement therapy was serially evaluated by the nephrology team through furosemide challenges and monitoring for acidosis, persistent electrolyte abnormalities, and signs of uremia. At a two-month follow-up with nephrology, CK level normalized to 124, and symptoms and electrolyte abnormalities were noted to resolve without indication for further intervention. Notably, risperidone was titrated down to 3 mg once a day.

## Discussion

The presentation of rhabdomyolysis can vary from mild enzyme elevation to severe renal disease with electrolyte abnormalities.^5^ Common complaints include muscle pains and weakness. Viral-induced rhabdomyolysis is clinically difficult to diagnose, as muscle pains and fatigue may also be attributed to the primary viral infection.^6^ A review of 27 cases of viral-induced rhabdomyolysis showed that 93% of patients reported myopathy symptoms within 1 week of the onset of viral pulmonary symptoms, emphasizing this clinical difficulty.^7^

In the present case, the etiology of rhabdomyolysis is most likely multifactorial and induced by an additive effect of this patient’s pharmacological and nonpharmacological predisposing factors. Although the patient did experience trauma due to the falls, the occurrence of weakness and dark urine prior to the falls suggested a more likely etiology. Other laboratory testing was negative, and clinical presentation, family history, and past medical history did not suggest autoimmune involvement. Similarly, serotonin syndrome, predisposed by serotonergic medications like tramadol, can also present with rhabdomyolysis; however, there were no other signs of serotonin syndrome, such as tremors, agitation, or autonomic instability. Risperidone has previously been implicated in antipsychotic-induced rhabdomyolysis; although antipsychotic medications have generally been shown to have an early failure pattern in inducing rhabdomyolysis, adverse effects can sometimes occur years after initiation.^8^ Given the timeline of rhabdomyolysis within days of Influenza A infection, the viral infection serves as a plausible trigger. In this case, there may have been a potentially synergistic effect involving the neuropsychiatric medications and the viral infection to trigger rhabdomyolysis.

Although the exact mechanism behind viral-induced rhabdomyolysis is unknown, several theories exist.^2^ First, the influenza virus may directly invade muscle cells.^9^ A second hypothesis proposes a cytokine-storm-like immune reaction to viral particles that results in collateral muscle damage.^2^ A third hypothesis states that viruses may release toxins into the bloodstream that cause muscle injury.^2^ A muscle biopsy was not pursued in this case, limiting the ability to investigate evidence for these theories in the present patient. Varying theories also exist for antipsychotic-induced rhabdomyolysis. One theory proposes that antipsychotics may increase skeletal muscle cell permeability through serotonin receptor antagonism, impairing the cell’s ability to uptake glucose.^10^ Another theory proposes that antipsychotic blockade of the nigrostriatal dopaminergic pathway causes unnecessary motor movements, which may result in high baseline CK levels.^11^

In the present case, the authors propose two theories. The patient’s use of risperidone may have lowered the rhabdomyolytic threshold, predisposing viral-induced rhabdomyolysis by the above mechanisms. Alternatively, the viral infection may have contributed to a systemic inflammatory state, exacerbating the muscle toxicity of risperidone and triggering antipsychotic-induced rhabdomyolysis. Given the rapid onset of rhabdomyolysis after Influenza A infection and the absence of recent medication regimen changes, the first theory may be more likely, with the viral infection acting as the final trigger in the setting of a lower baseline rhabdomyolysis threshold. Literature describing rhabdomyolysis in patients with multiple potential etiologies is scarce, and our report emphasizes the need for larger-scale investigation of an additive effect. These hypotheses are limited by the small sample size of our report and require further validation with reproducible models.

Evidence of muscle necrosis is the ideal diagnostic criterion for rhabdomyolysis, although diagnosis may be confirmed with a CK level over 1000 U/L or five times the upper limit of normal.^5,7^ In the present case, CK level at presentation was over 60,000 U/L. Monitoring CK levels may help predict prognosis and disease progression, as higher levels may predict renal dysfunction, need for renal replacement therapy, and severity of pulmonary infection.^12^ Although rhabdomyolysis is known to be a rare complication of Influenza A, studies suggest that this complication may be underreported. A study of the 2009 influenza epidemic reports elevated CK levels in up to 62% of influenza-infected patients, although this trend cannot necessarily be generalized to seasonal viral illnesses.^13^

Early recognition and aggressive treatment are needed to avoid renal failure. Treatment must incorporate antiviral therapy (oseltamivir in the present case) and fluid supplementation to dilute the heme pigment and prevent further renal insult.^3^ A urine output rate of 200–300 mL/h can be used as a benchmark for the optimal rate of fluid administration.^3^ Notably, caution must be exercised to avoid fluid overload, which developed in the present patient as pulmonary edema (Figure 1) and necessitated additional management to avoid further disease morbidity. In patients with respiratory comorbidity (such as Influenza A infection), fluid overload can be a life-threatening complication, and clinicians’ threshold to address fluid overload concerns must be appropriately adjusted. This may have been avoided in this case by increasing IV fluids from a lower initial rate as opposed to decreasing IV fluids from a higher initial rate.

In addition, sodium bicarbonate is often used to address acidosis and electrolyte abnormalities if alkaline diuresis is indicated.^2^ Although rhabdomyolysis in this case resolved spontaneously with relatively conservative management, worsening renal insult may eventually necessitate renal replacement therapy. Electrolytes should also be monitored. In the present case, hyperkalemia developed on day 4 of hospital stay and was managed with potassium diuresis and insulin-induced intracellular potassium shift. Clinicians must include rhabdomyolysis in the differential in patients with viral illness and muscle pains, especially when comorbidities, such as the use of antipsychotic or serotonergic medications, are present.

## Conclusions

Influenza A-associated rhabdomyolysis is a rare, devastating complication. All patients with Influenza A complaining of severe muscle pain should be evaluated with CK measurement, but a higher threshold of suspicion for rhabdomyolysis should be exercised in patients with Influenza A who have other rhabdomyolysis-predisposing factors, such as use of antipsychotics or serotonergic agents. In patients with viral illness taking rhabdomyolytic agents, clinicians may consider decreasing the dosages and advising patients to increase hydration to decrease the risk of rhabdomyolysis. In patients confirmed to have rhabdomyolysis, serial CK lab measurements can help monitor disease progression and predict prognosis. Electrolyte abnormalities should be promptly managed to prevent further deterioration. Clinicians must take a proactive approach in suspecting influenza-induced rhabdomyolysis and managing with antiviral therapy and fluid resuscitation, with a goal of urine output of 200–300 mL/h. However, caution must be exercised to balance fluid resuscitation with the risk of fluid overload, especially in patients with respiratory comorbidities like viral infection.

## Supplemental Material

sj-docx-1-sco-10.1177_2050313X251392105 – Supplemental material for Rhabdomyolysis secondary to Influenza A infection in a patient using antipsychotic and serotonergic agents: A case report Supplemental material, sj-docx-1-sco-10.1177_2050313X251392105 for Rhabdomyolysis secondary to Influenza A infection in a patient using antipsychotic and serotonergic agents: A case report by Rahul S. Nanduri, Noah Karnath, Alekhya Gurram, Arkoon Ali and Bernard Karnath in SAGE Open Medical Case Reports
