# Supplementary material for: Rhabdomyolysis secondary to Influenza A infection in a patient using antipsychotic and serotonergic agents: A case report
Source: SAGE Open Med Case Rep. 2025 Nov 1;13:2050313X251392105. doi: 10.1177/2050313X251392105 (PMC12580520; doi:10.1177/2050313X251392105)
Supplement: sj-docx-1-sco-10.1177_2050313X251392105 – Supplemental material for Rhabdomyolysis secondary to Influenza A infection in a patient using antipsychotic and serotonergic agents: A case report [file sj-docx-1-sco-10.1177_2050313X251392105.docx]

#
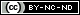

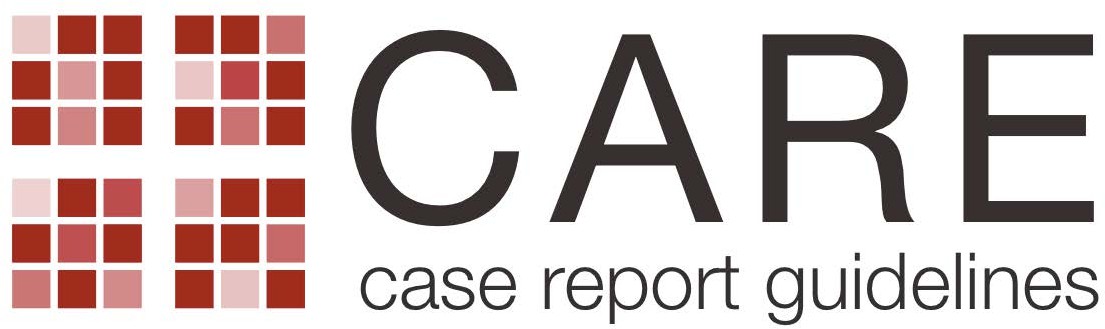
CARE Checklist of information to include when writing a case report

**Topic Item Checklist item description Reported on Line**

**Title 1** The diagnosis or intervention of primary focus followed by the words “case report” . . . . . . . . . . . . . . . . . . 1,2

**Key Words 2** 2 to 5 key words that identify diagnoses or interventions in this case report, including "case report" 12, 13

**Abstract**

**(no references)**

**3a** Introduction: What is unique about this case and what does it add to the scientific literature? 23-33

**3b** Main symptoms and/or important clinical findings . . . . . . . . . . . . . . . . . . . . . . . . . . . . . . . . . . . . . . . . . . . . . . . . . . . 18-26

**3c** The main diagnoses, therapeutic interventions, and outcomes 26-33

**3d** Conclusion—What is the main “take-away” lesson(s) from this case? 26-33

**Introduction 4** One or two paragraphs summarizing why this case is unique (**may include references**) 36-65

**Patient Information 5a** De-identified patient specific information 68-71

**5b** Primary concerns and symptoms of the patient 71-90

**5c** Medical, family, and psycho-social history including relevant genetic information 67-71

**5d** Relevant past interventions with outcomes 71-90

**Clinical Findings**

**Timeline**

**Diagnostic Assessment**

**Therapeutic Intervention**

**Follow-up and Outcomes**

1. Describe significant physical examination (PE) and important clinical findings 93-96
2. Historical and current information from this episode of care organized as a timeline 71-96, Figure 3

**8a** Diagnostic testing (such as PE, laboratory testing, imaging, surveys). 96-99

**8b** Diagnostic challenges (such as access to testing, financial, or cultural) N/A

**8c** Diagnosis (including other diagnoses considered) 99-102

**8d** Prognosis (such as staging in oncology) where applicable 138-141 ____

**9a** Types of therapeutic intervention (such as pharmacologic, surgical, preventive, self-care) . . . . . . . . . . . . . . . . . 99-102

**9b** Administration of therapeutic intervention (such as dosage, strength, duration)………………………………………..99-102, 103-105, 106-109, 128-131, 139-141

**9c** Changes in therapeutic intervention (with rationale) 103-104, 106-107, 107-109, 128-130, 139-142

**10a** Clinician and patient-assessed outcomes (if available) 139-142

**10b** Important follow-up diagnostic and other test results 150-153

**10c** Intervention adherence and tolerability (How was this assessed?) 148-153

**10d** Adverse and unanticipated events 109-110

**Discussion 11a** A scientific discussion of the strengths AND limitations associated with this case report 216-220

**11b** Discussion of the relevant medical literature **with references** 374-418

**11c** The scientific rationale for any conclusions (including assessment of possible causes) 189-241

**11d** The primary “take-away” lessons of this case report (without references) in a one paragraph conclusion 324-353

**Patient Perspective 12** The patient should share their perspective in one to two paragraphs on the treatment(s) they received . . . . Unable to reach patient

**Informed Consent 13** Did the patient give informed consent? Please provide if requested . . . . . . . . . . . . . . . . . . . . . . . . . . . . . . . . . . . . . . **Yes
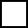
** **No
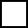
**
